# Cost-Effectiveness Analysis of Tdap in the Prevention of Pertussis in the Elderly

**DOI:** 10.1371/journal.pone.0067260

**Published:** 2013-09-03

**Authors:** Lisa J. McGarry, Girishanthy Krishnarajah, Gregory Hill, Michelle Skornicki, Narin Pruttivarasin, Cristina Masseria, Bhakti Arondekar, Stephen I. Pelton, Milton C. Weinstein

**Affiliations:** 1 OptumInsight, Cambridge, Massachusetts, United States of America; 2 GlaxoSmithKline, Philadelphia, Pennsylvania, United States of America; 3 Boston University, Boston, Massachusetts, United States of America; 4 Harvard University, Harvard School of Public Health, Boston, Massachusetts, United States of America; Erasmus University Rotterdam, Netherlands

## Abstract

**Objectives:**

Health benefits and costs of combined reduced-antigen-content tetanus, diphtheria, and pertussis (Tdap) immunization among adults ≥65 years have not been evaluated. In February 2012, the Advisory Committee on Immunization Practices (ACIP) recommended expanding Tdap vaccination (one single dose) to include adults ≥65 years not previously vaccinated with Tdap. Our study estimated the health and economic outcomes of one-time replacement of the decennial tetanus and diphtheria (Td) booster with Tdap in the 10% of individuals aged 65 years assumed eligible each year compared with a baseline scenario of continued Td vaccination.

**Methods:**

We constructed a model evaluating the cost-effectiveness of vaccinating a cohort of adults aged 65 with Tdap, by calculating pertussis cases averted due to direct vaccine effects only. Results are presented from societal and payer perspectives for a range of pertussis incidences (25–200 cases per 100,000), due to the uncertainty in estimating true annual incidence. Cases averted were accrued throughout the patient 's lifetime, and a probability tree used to estimate the clinical outcomes and costs (US$ 2010) for each case. Quality-adjusted life-years (QALYs) lost to acute disease were calculated by multiplying cases of mild/moderate/severe pertussis by the associated health-state disutility; QALY losses due to death and long-term sequelae were also considered. Incremental costs and QALYs were summed over the cohort to derive incremental cost-effectiveness ratios. Scenario analyses evaluated the effect of alternative plausible parameter estimates on results.

**Results:**

At incidence levels of 25, 100, 200 cases/100,000, vaccinating adults aged 65 years costs an additional $336,000, $63,000 and $17,000/QALY gained, respectively. Vaccination has a cost-effectiveness ratio less than $50,000/QALY if pertussis incidence is >116 cases/100,000 from societal and payer perspectives. Results were robust to scenario analyses.

**Conclusions:**

Tdap immunization of adults aged 65 years according to current ACIP recommendations is a cost-effective health-care intervention at plausible incidence assumptions.

## Introduction

Although the incidence of pertussis substantially declined immediately following the introduction of reduced-antigen-content tetanus, diphtheria and acellular pertussis (Tdap) vaccination recommendations in 1997 and 2005, thereafter rates have steadily increased to the point that pertussis causes a significant burden of disease in the US [1], [2].

In 2005, the Advisory Committee on Immunization Practices (ACIP) recommended that adolescents 11–18 years of age (preferably at age 11–12 years) and adults aged 19–64 years should receive a single dose of Tdap vaccine, instead of the standard tetanus and reduced-antigen-content diphtheria (Td) decennial booster [1]. Subsequently, in October 2010, ACIP recommended expanded use of Tdap, advising that children aged 7 through 10 years not previously fully vaccinated against pertussis receive a single dose of Tdap, and that adults ≥65 years who have close contact with an infant receive a single dose of Tdap if they have not previously received the booster [3]. Despite recommendations, Tdap coverage remains relatively low at 56% for the adolescent booster and <6% for the adult booster as of 2010 [4].

Since 2007, pertussis incidence has been increasing and continues to remain higher than in the 1990 s [2]. It is unclear whether this recent increase in incidence reflects changes in the epidemiology or in disease reporting. Although pertussis is nationally notifiable, many cases are believed to go unrecognized and/or unreported [5], so the true incidence of disease is uncertain. Serologic studies have suggested that the true incidence in adults may be more than 100 times the incidence reported to the Centers for Disease Control and Prevention (CDC) [6], [7]. Despite this uncertainty, current evidence clearly suggests that recent vaccination practices have not completely controlled the risk of pertussis infection.

In February 2012 ACIP extended the age range for its recommendation of administration of a single dose Tdap vaccine to all adults (including those ≥65 years) and removed limits on the interval since last tetanus or diphtheria toxoid-containing vaccine, reflecting the belief that additional Tdap vaccination in the population aged ≥65 may yield additional benefits in disease prevention [8].

Two Tdap vaccines are licensed in the US, *Boostrix®* and *Adacel®*. *Boostrix®*, manufactured by GlaxoSmithKline, is indicated for individuals 10 years of age and older. *Adacel®*, manufactured by Sanofi Pasteur, is indicated for individuals 11–64 years of age. The current analysis was conducted to assess the impact of the new ACIP recommendation for Tdap vaccination in the population aged ≥65 years, using a static model of a cohort of adults vaccinated at 65 years.

### Model Overview and Structure

**Figure 1:**
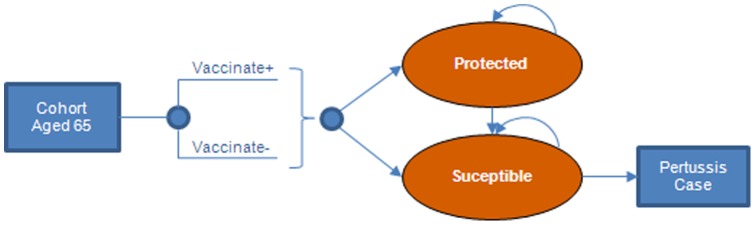
Vaccination, waning protection and pertussis cases in a cohort aged 65 years.

**Figure 2:**
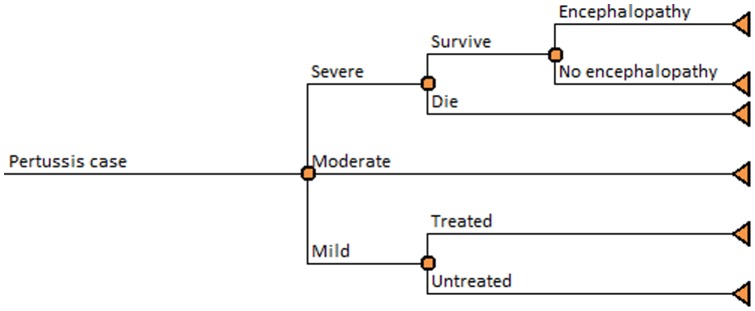
Economic model.

The economic model (Figure 1–2) was constructed in Microsoft Excel®. The model assesses the cost-effectiveness of vaccinating a cohort of adults aged 65 years with *Tdap*, by calculating pertussis cases averted due to direct vaccine effects only. Results were evaluated from the societal and the payer perspectives. The societal perspective, which is consistent with the Reference Case recommendations of the US Panel on Cost-Effectiveness in Health and Medicine [9], considers both direct medical costs of pertussis treatment and direct non-medical costs of patient 's time in hospital. The payer perspective considers only direct medical-care costs.

The cost-effectiveness of the intervention was estimated by calculating the incremental costs and outcomes associated with a strategy of vaccinating adults aged 65 years with Tdap versus Td. The same proportion (10%) of the 65-year-old cohort was vaccinated under both strategies. Tdap vaccinated and Td vaccinated cohorts were followed in the model for 35 years using annual cycles (adjusted for age-specific mortality [10]). A scenario analysis was conducted using a 10-year model time horizon.

We estimated the yearly probability of protection from pertussis, assuming that vaccine protection wanes at a constant annual rate, (i.e., following an exponential distribution), to determine the proportion of vaccinated individuals who remained protected at each age. Cases averted each year were calculated as the Tdap vaccine efficacy multiplied by the number of individuals protected at each age, multiplied by the incidence of pertussis. Cases averted were accrued throughout the remaining lifetime of the cohort. Cases were stratified by severity, where all severe and moderate cases were treated and a proportion of mild cases were untreated (Figure 2).

The model was used to estimate the total number of pertussis cases avoided, quality-adjusted life-years (QALYs) gained, and total direct medical and non-medical costs averted.

### Model Inputs

Model inputs were obtained from publicly-available data and peer-reviewed publications listed in Table 1.

**Table 1:** Model inputs and sources for adults aged 65 years.

Parameter	Value	Scenario Analysis Values	PSA Distribution (Values)	Source
Disease Inputs				
Incidence Range (per 100,000)	25		Uniform (Range: 25/ 100,000 – 200/100,000)	Assumptions based on CDC Morbidity and Mortality Weekly Report 2009 (Vol.58/No.53), California Department of Public Health Pertussis Report 2011
	50			
	100			
	150			
	200			
Coverage	10%			Assumption
Tdap Efficacy	89%	77%	Lognormal (95% CI: 77.6%–94.6%)	Schmitt 1996
Duration of Protection	8 years	6, 10 years	Uniform (Range: 4–10 years)	De Vries 2010
Population adults 65 years	2,592,176			U.S. Census Bureau 2010
Duration of Illness	87 days	56 days	Lognormal (95% CI: 41.1–101.9)	Schmitt 1996
Duration of most severe symptoms	2 weeks	1 week		Assumption
Proportion of cases that are severe	12.0%	Varies by incidence (12.0–14.1%)	Beta: (SE: 1.04%)	Cortese 2007
Proportion of cases that are moderate	74.0%	Varies by incidence (74.0–84.7%)		Residual of severe and mild disease
Proportion of cases that are mild	14.0%	Varies by incidence (1.1–14.0%)		Cortese 2007
Probability of encephalopathy (permanent sequelae) among severe cases	0.47%	0%		Caro 2005
Probability of death among severe cases	0.86%	0.43%		Caro 2005
Proportion of unreported cases that receive medical care	70.7%			Molinari 2007
Economic Inputs				
Additional Cost of Tdap Vaccine*	$18.10	$16.16, $17.84		CDC vaccine price list 2010, Lee 2007
Cost of Treating Mild Case of Pertussis	$99.22	$0		Physicians ' Fee and Coding Guide 2010, Red Book 2010, McDowell 2009
Cost of Treating Moderate Case of Pertussis	$203.13	$443.38		Lee 2000
Cost of Treating Severe Case of Pertussis	$7,221.97	$443.38	Lognormal (95% CI: $5,839– $37,572)	O 'Brien 2005
Days of work lost due to hospitalization, reported cases	0.55			O 'Brien 2005, BLS Curent Population Survey 2010
Value of lost productivity per day	$114.30	$607.95**	Gamma (SE: $23.42)	Bureau of Labor Statistics 2010; Lee 2004
Utility Inputs				
Disutility of pertussis with mild symptoms	0.10	0.09, 0.08	Varied as fixed multiple of severe	De Vries 2010
Disutility of pertussis with moderate symptoms	0.15	0.14, 0.12	Varied as fixed multiple of severe	De Vries 2010
Disutility of pertussis with severe symptoms	0.19	0.17, 0.15	Beta (SE: 0.039)	De Vries 2010
Disutility of encephalopathy	0.20	0.18, 0.16	Beta (SE: 0.007)	Wells 2004

* Vaccine cost was estimated as the incremental cost of Tdap versus Td and included drug acquisition cost and cost of vaccine adverse events (Lee 2007).

** Cost is lost productivity per case and is applied to moderate and severe cases only.

PSA = probabilistic sensitivity analysis.

#### Pertussis Incidence

In the adult population, pertussis is widely under-recognized and underreported. While the available but limited studies of pertussis incidence in adults report a wide range of incidence between 66–500 cases/100,000 [11], fewer than 5 cases/100,000 are reported to the CDC [2]. Recent outbreaks in California and Washington State provided incidence estimates that ranged widely from no cases to more than 400/100,000 in selected counties or age groups [12], [13]. Due to this uncertainty in the true pertussis incidence in adults ≥65 years, outcomes were assessed at different incidence rates (25, 50, 100, 150, and 200 cases/100,000) consistent with previously published models [14]. Incidence rates are also consistent with the rate used in a cost-effectiveness model of Tdap in adults aged ≥65 years conducted by the CDC (104 cases/100,000) [11].

#### Vaccination and Disease Outcomes

**Figure 3:**
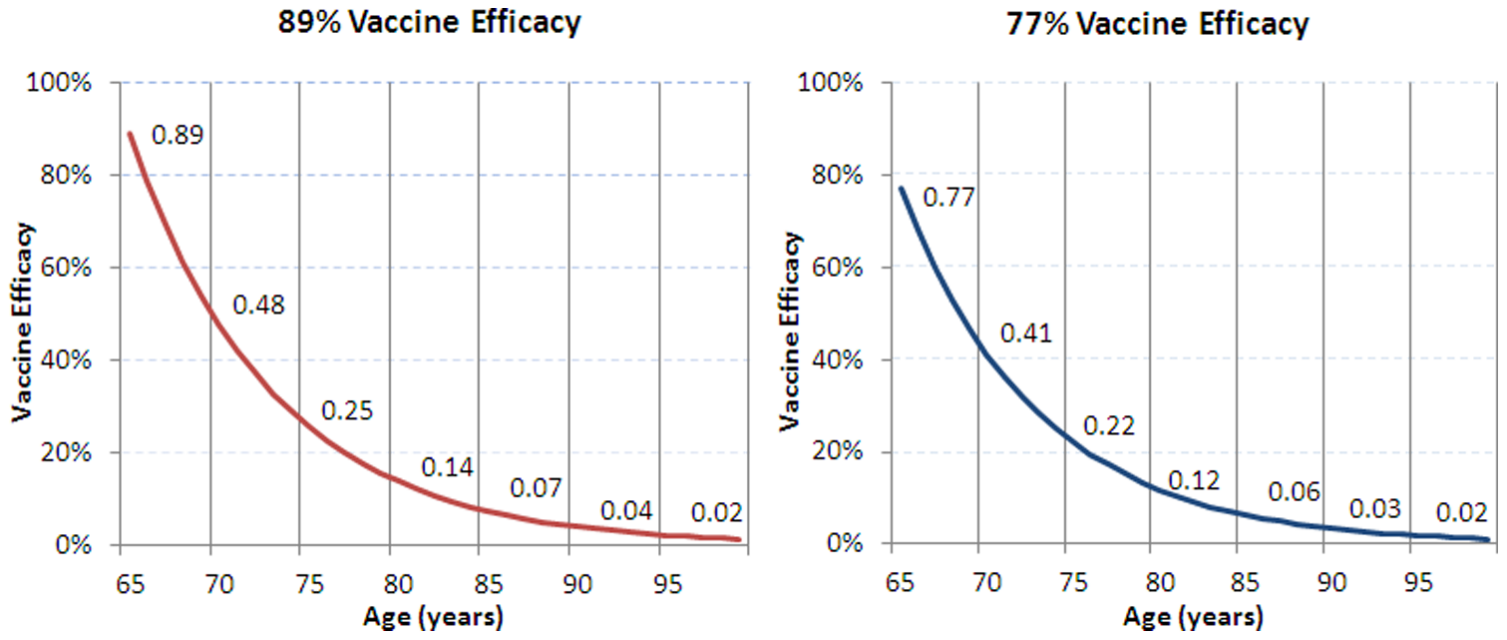
*Tdap* waning vaccine efficacy.

We assumed that 10% of the population aged 65 years old will receive Tdap in the intervention group and Td in the comparison group; this is consistent with an equal portion of the population becoming eligible for the decennial Td booster each year. Tdap vaccine efficacy in the base case was estimated to be 89%. *Tdap* efficacy was not directly measured but estimated via immunobridging to an infant DTaP efficacy study (*Infanrix*®, GlaxoSmithKline) [15]. In the study used for efficacy bridging, vaccine efficacy was shown to be 89%. A scenario analysis was conducted assuming 77% efficacy based on the lower limit of the 95% confidence interval in the DTaP vaccine efficacy trial [13]. Waning immunity is assumed to occur at a constant rate, with a mean duration of protection of 8 years (Figure 3) [16]. Scenario analyses were conducted with alternative duration of protection estimates of 6 and 10 years.

We classified each case generated by the model as severe (requiring hospitalization), moderate (non-hospitalized cases with symptoms of paroxysmal cough), or mild (non-hospitalized cases without paroxysmal cough). In the base-case, the distribution of cases by severity was assumed to be the same for all incidence levels. The proportion of severe cases was based on hospitalization rates reported to CDC among reported cases [5]. The proportion of mild cases was based on the percentage of reported cases without paroxysmal cough [5]. The proportion of moderate cases was assumed to be those not classified as severe or mild. A scenario analysis was conducted in which the distribution of cases by severity was dependent on overall incidence level, similar to a published cost-effectiveness study of pertussis vaccination in adults aged 20–64 years [12].

Patients with severe pertussis are at risk of encephalopathy or death. We used probability of encephalopathy to estimate loss of quality of life associated with long-term disability in addition to the loss of QALYs due to death. We estimated that 33% of encephalitis cases would result in permanent sequelae, consistent with encephalopathy [17]. The probabilities of encephalopathy or death among severe cases were based on 1997–2000 data for reported hospitalized cases [16]. an alternative assumption of no cases of encephalopathy was also included as a scenario analysis.

A proportion of mild cases was assumed to receive medical treatment; due to the limited available data, we assumed that the proportion of mild pertussis cases seeking treatment is the same as the proportion of influenza patients with an outpatient visit [18].

#### Utilities

Utility values, preference-based measures of quality-of-life ranging from 0 to 1, that are used to calculate QALYs, were obtained from the published literature [15], [19]. Disutilities of mild, moderate, and severe pertussis and encephalopathy were used to estimate the QALY loss associated with acute pertussis by multiplying the associated decrement by the time spent in the health state (Table 1). Total duration of illness (87 days), obtained from published literature [15], was assumed to be the same for severe, moderate, and mild diseases. A mild case received the mild symptom disutility for the full length of illness. Moderate cases received two weeks of moderate symptom disutility followed by mild symptom disutility for the remaining days of illness. A severe case received two weeks of severe symptom disutility followed by mild symptom disutility for the remaining days of illness. In addition, patients with encephalopathy were assumed to receive the disutility for encephalopathy for the remainder of their lifetime. Loss of utility due to death was based on age-specific US population norms estimated by Hanmer and colleagues, which reflect decreasing health-related quality-of-life at older ages [20]. Total QALYs lost due to death or encephalopathy were calculated as the discounted (at 3% per annum) present values of the lost utility streams over the remaining expected lifetime, based on age-specific life expectancy [10] at the time of the event.

#### Costs

Direct medical costs included vaccine costs and acute costs of treating severe, moderate, and mild pertussis (Table 1). Vaccine cost was obtained from the CDC vaccine price list [21] based on the private incremental cost of Tdap versus Td. We assumed that adults receive the private price and were administered Tdap in place of their decennial Td booster; therefore no additional administration fee was considered, and only the marginal acquisition cost of Tdap versus Td was counted. Costs of vaccine adverse events, weighted by the probabilities of occurrence (incremental to Td adverse events), were also included [12]. Scenario analyses were conducted assuming public and mixed public and private (weighted by the proportion who receive the vaccine at public cost) [22] incremental vaccine costs. As appropriate, costs were converted to 2010 U.S. dollars using the medical component of the Consumer Price Index [23].

The cost of treating a mild case was estimated as the cost of an antibiotic regimen (azithromycin) [24], [25] and the cost of a level 2 physician visit [26]. Cost of treating a moderate case was based on a prospective study of pertussis morbidity among families in a community setting, which included physician visits, emergency room visits, laboratory, and medication costs [27]. The cost of treating a severe case of pertussis was based on total hospitalization cost, including all accommodations (e.g., routine, intensive care unit, nursery), ancillary (e.g., pharmacy, laboratory, imaging), and physician services [28]. Hospitalization costs were obtained from a published study of pertussis hospitalizations and included hospitalized cases due to pertussis complications such as pneumonia, convulsions, apnea, encephalopathy, and acute respiratory distress, failure, or arrest [23]. Because the acute cost of encephalopathy was included in the hospitalization cost, it was not estimated separately. No long-term medical care costs were considered, including survivor costs or long-term costs of encephalopathy.

Direct non-medical costs included lost productivity from time missed from work due to hospitalization, and therefore applied only to severe cases (Table 1). We estimated work loss based on published estimates of hospital length of stay combined with estimates of age-specific employment rates and mean wage [23], [29], [30]. We did not consider the cost of productivity loss due to days spent at home with illness, or lifetime productivity loss among fatalities.

#### Analyses

Cumulative lifetime costs and QALYs were estimated for each vaccine strategy. Cumulative costs are calculated as the sum of costs of vaccination and associated adverse events, and the costs of treating pertussis, including treatment costs and work loss due to hospitalization; QALYs lost are calculated as the sum of losses due to acute disease and losses to death or encephalopathy. Costs incurred and QALYs gained after the first year of the model were discounted at 3% per year. Incremental cost-effectiveness ratios (ICERs) were calculated by dividing the incremental cost of the Tdap strategy, compared to Td, by its incremental benefit in terms of additional QALYs gained (i.e., reduction in QALYs lost).

In addition to the lifetime horizon, we examined results at 10 years post-vaccination. To assess the model results under different parameter assumptions, scenario analyses were conducted with increased vaccine price, reduced efficacy, reduced duration of vaccine protection, reduced proportion with severe symptoms, reduced mortality, reduced duration of symptoms, reduced duration of moderate or severe disutility, no encephalopathy, and reduced overall QALY decrements (by 10% and 20%). A scenario analysis was also conducted using estimated direct medical and direct non-medical treatment costs from alternative published sources [12], [31].

A second-order probabilistic Monte Carlo sensitivity analysis (PSA) was conducted to assess the sensitivity of study findings to overall parameter uncertainty, particularly with regard to vaccine efficacy and waning, pertussis incidence and duration, costs, and utilities. Uncertainty in key model parameters was characterized by probability distributions with means equal to the base-case estimates. Assumptions used in sensitivity analyses are summarized in Table 1. Tdap efficacy was varied by varying the relative risk of pertussis versus no vaccination based on the log-normal distribution, Parameters for which only a range was available were assumed to follow either a uniform or a lognormal distribution.

## Results

Depending on the incidence assumption, one-time vaccination of a 10% cohort of adults aged 65 years is expected to result in approximately 400 to 3,300 total pertussis cases avoided over the lifetime of the vaccinated cohort (Table 2). Predicted total direct medical cost savings ranged from approximately $360,000 at an incidence of 25/100,000 to $2.9 million for an incidence of 200/100,000; although the majority of cases prevented were of mild and moderate severity, prevention of severe cases accounted for >80% of the cost savings at all incidence levels (Table 3). Predicted savings from direct non-medical costs were estimated to comprise about 1% of the total cost savings; therefore, results from the societal and payer perspectives did not differ greatly. The estimated $4.69 million in vaccination costs was only partially offset by cost savings from disease prevention. Prevention savings ranged from less than 10% of vaccine costs at 25/100,000 to two-thirds of the vaccine cost at 250/100.000; the vaccination program was not cost-saving at any incidence level examined.

**Table 2:** Pertussis cases avoided by vaccinating 10% of US population aged 65 years.

	Incidence (per 100,000)				
Case Type	25	50	100	150	200
Severe cases	49.3	98.6	197.1	295.7	394.2
Deaths	0.4	0.8	1.7	2.5	3.4
Encephalopathy	0.2	0.5	0.9	1.4	1.8
Moderate Cases	303.9	607.8	1,215.5	1,823.3	2,431.0
Mild Cases	57.5	115.0	230.0	344.9	459.9
Treated	40.6	81.2	162.5	243.7	325.0
Untreated	16.9	33.7	67.5	101.2	134.9
**Total Cases**	**410.6**	**821.3**	**1,642.6**	**2,463.9**	**3,285.2**

**Table 3:** Incremental costs from vaccinating 10% of US population aged 65 years.

	Incidence (per 100,000)				
Cost Type	25	50	100	150	200
**Direct Medical Cost (Savings)**					
Mild treated	($3,467)	($6,934)	($13,867)	($20,801)	($27,734)
Moderate	($53,088)	($106,177)	($212,354)	($318,531)	($424,707)
Severe	($306,083)	($612,167)	($1,224,334)	($1,836,500)	($2,448,667)
Total	($362,639)	($725,277)	($1,450,554)	($2,175,832)	($2,901,109)
**Direct Non-Medical (Lost Productivity) Cost (Savings)**					
Mild treated	($0)	($0)	($0)	($0)	($0)
Moderate	($0)	($0)	($0)	($0)	($0)
Severe	($3,102)	($6,205)	($12,409)	($18,614)	($24,819)
Total	($3,102)	($6,205)	($12,409)	($18,614)	($24,819)
**Direct Medical and Direct Non-Medical Cost (Savings)**	($365,741)	($731,482)	($1,462,964)	($2,194,446)	($2,925,928)
**Vaccination Cost**	$4,691,839	$4,691,839	$4,691,839	$4,691,839	$4,691,839
**Total Costs Including Direct Non-Medical Cost**	$4,326,098	$3,960,357	$3,228,876	$2,497,393	$1,765,911
**Total Costs Excluding Direct Non-Medical Cost**	$4,329,200	$3,966,562	$3,241,285	$2,516,007	$1,790,730

The cost-effectiveness results in terms of incremental cost per case averted and per QALY gained from the societal perspective are presented in Table 4. Introducing one-time Tdap vaccination among a cohort of 10% of U.S. residents aged 65 years results in a cost per case averted ranging from $10,500 to $538 and cost per QALY gained ranging from $336,000 to $17,100, depending on the incidence level, from the societal perspective. Analyses from the payer perspective yielded similar results. Vaccinating adults aged 65 years has an ICER of less than $50,000 per QALY gained if pertussis incidence in adults is at least 116 cases/100,000, from both the societal and payer perspectives.

**Table 4:** Incremental cost per case averted and per QALY gained (89% efficacy), societal perspective.

Incidence (cases/ 100,000)	Cases averted	Discounted Incremental costs	Discounted Incremental QALYs	Incremental Cost/Case averted	Incremental Cost/QALY gained
25	410.65	$4,326,102	12.87	$10,535	$336,108
50	821.29	$3,960,366	25.74	$4,822	$153,846
100	1,642.59	$3,228,893	51.48	$1,966	$62,716
150	2,463.88	$2,497,420	77.23	$1,014	$32,339
200	3,285.17	$1,765,947	102.97	$538	$17,150

A series of scenario and sensitivity analysis were conducted to test various assumptions in the model (Table 5). For the scenario assuming 77% Tdap efficacy, cost per QALY gained from the societal perspective increased by 17% from the base-case at an incidence of 25 per 100,000 and by 41% at an incidence of 200 per 100,000. When duration of protection was decreased, incremental cost-effectiveness ratios (ICERs) per QALY gained increased by 20% to 51% from the base-case societal perspective, depending on incidence levels. Increasing the duration of protection to 10 years caused the ICER to decrease from the base-case societal perspective by 12% at an incidence of 25 per 100,000 and by 31% at an incidence of 200 per 100,000. Results were most sensitive to the model time horizon, vaccine characteristics (vaccine efficacy, duration of protection), and the outcomes associated with severe cases (proportion severe, mortality, utility decrement and duration of symptoms). The cost-effectiveness ratio of vaccinating adults aged 65 years remained less than $50,000/QALY gained at an incidence level of 150 cases/100,000 across all scenario analyses from the societal perspective except when we reduced the proportion of severe case by 50%, where it was $51,800/QALY gained.

**Table 5:** Scenario analyses, societal perspective.

	Incremental Cost per QALY gained				
Scenario Analysis	Incidence 25/ 100,000	Incidence 50/ 100,000	Incidence 100/ 100,000	Incidence 150/ 100,000	Incidence 200/ 100,000
**Base case Scenario**	**$336,108**	**$153,846**	**$62,716**	**$32,339**	**$17,150**
Model horizon 10 years	$393,277	$182,684	$77,387	$42,288	$24,739
Vaccine efficacy 77%	$392,916	$182,250	$76,917	$41,806	$24,251
Duration of protection 6 Years	$404,039	$187,945	$79,897	$43,882	$25,874
No cases of encephalopathy	$348,822	$159,666	$65,088	$33,562	$17,799
Mortality decreased by 50%	$386,188	$176,769	$72,060	$37,157	$19,705
Reduce proportion of severe cases by 50%	$409,440	$194,867	$87,580	$51,818	$33,937
Duration of protection 10 years	$294,973	$133,178	$52,280	$25,314	$11,831
Public price for Tdap vs. Td ($16.16)	$297,037	$134,311	$52,948	$25,827	$12,266
Mixed public and private price* ($17.84)	$330,871	$151,228	$61,406	$31,466	$16,495
QALY decrement: −10%	$362,950	$166,133	$67,724	$34,921	$18,519
QALY decrement: −20%	$394,452	$180,552	$73,602	$37,952	$20,127
Duration of symptoms decreased from 86 to 56 days	$438,013	$200,491	$81,730	$42,143	$22,350
Duration of moderate and severe symptoms decreased from 2 to 1 week	$344,757	$157,805	$64,329	$33,171	$17,591
Costing using Lee 2004 and 2007	$339,388	$157,126	$65,995	$35,618	$20,430

* Mixed public and private incremental vaccine cost was calculated by weighing the public and private vaccine cost by the proportion of those who receive the vaccine at public cost.

**Figure 4:**
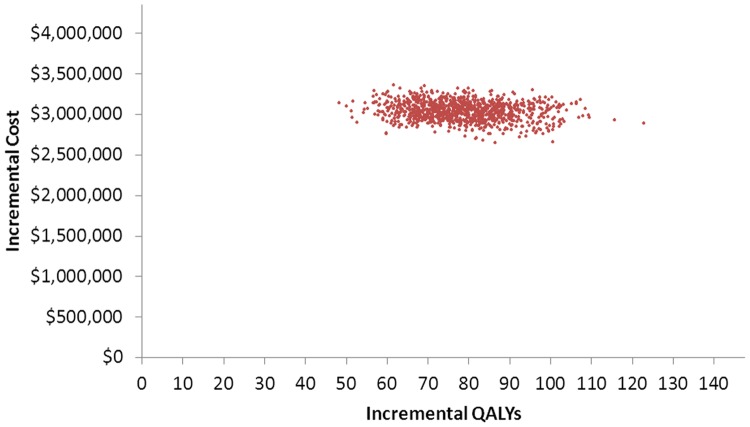
Probabilistic sensitivity analysis, societal perspective.

The PSA using 1,000 simulations resulted in a distribution of incremental costs and QALYs gained with a mean ICER of $39,500 and 95% of ICER values falling between $28,900 and $53,400 (Figure 4). In all simulations, both incremental costs and QALYs were higher for the Tdap versus Td vaccination program.

## Discussion

We constructed a cohort model to estimate the cases averted and cost implications of replacing the decennial Td vaccine with Tdap vaccine, in adults aged 65 years.

Model results were generated for incidence levels ranging from 25 cases/100,000 to 200 cases/100,000; vaccinating 10% of adults aged 65 years resulted in an incremental cost per case averted of approximately $500 to $10,500 and an incremental cost per QALY gained of $17,200 to $336,100 from the societal perspective, depending on incidence assumptions.

The findings of our study are based on a number of assumptions, and limitations of the model should be considered when interpreting results. First, the model considers only the benefits of vaccination to the vaccinated individuals and does not evaluate indirect effects (herd effects) from reduced transmission of pertussis from protected individuals; A recently published analysis by Coudeville and colleagues of vaccination among adults age <65 using a dynamic transmission model [24], suggests that including these effects would likely have made the results more favorable for Tdap boosting. In addition, the model evaluates only a 65-year-old cohort, whose outcomes cannot be extrapolated to other age groups. A further study limitation is that data used to estimate vaccine effectiveness were taken from immunogenicity trials of DTaP in a pediatric population and may not reflect the immune response expected in the 65 year old population. Scenario analyses showed the model was sensitive to lower efficacy assumptions, but results remained favorable at similar incidence levels as with the base case efficacy. We used data specific to the 65+ population in model estimation where available; however our estimates for vaccine efficacy, duration of symptoms and utility were derived from studies in younger populations and may not reflect the experience of those aged 65+. We also note that the population aged 65+ is treated as homogeneous and we did not model natural immunity or potential changes in disease susceptibility and outcome due to immunosenescence or comorbidity as individuals age. Therefore, to the extent that vaccination at age 65 shifts disease to older age groups with worse outcomes, our analysis may overstate the benefits of the vaccination program.

Pertussis incidence data showed a decreasing pattern after the 2005 adult and adolescent vaccination recommendation, followed by an apparent increase in incidence thereafter [2]. The reason for this recent increase in incidence is unclear and may represent changes in reporting rather than in true incidence. Estimates from recent outbreaks have shown great variability in reported cases. Surveillance data from a California outbreak in 2010 reported 23.4 cases/100,000 residents, with reported rates ranging from 435.0/100,000 for infants aged <6 months to 5.2 reported cases/100,00 among those aged >65 [12]. In the recent pertussis outbreak in Washington State, through June 2012 there were 39.3 reported pertussis cases/100,000 persons, with incidence ranging from 4.9 cases per 100,000 to 425.8 cases per 100,000, across counties reporting cases [13]. Due to this uncertainty in the true incidence, we chose to assess outcomes across a range of incidence values for our analyses. We note that in doing so, we chose incidence levels that are all higher than CDC reported cases. Because our severity estimates are based on reported cases, we may be overestimating the severity of all pertussis cases if unreported cases are milder than reported. We conducted sensitivity analyses in which we decreased the proportion of severe cases by 50% and found that results were indeed less favorable, although still in the cost-effective range at higher incidence assumptions. We recognize, therefore, that to the extent our estimates over- or under-state the severity of pertussis cases, we may be over-or under-estimating the potential savings in pertussis treatment costs with the Tdap vaccination program.

In base-case analyses, we did not consider either the lifetime future healthcare costs for diseases unrelated to pertussis or the net lifetime consumption costs among pertussis survivors, as suggested by Meltzer [32]. Inclusion of future unrelated healthcare costs is considered to be optional and not part of the reference case in the US or other countries outside Sweden; inclusion of future consumption is not recommended in any current guidelines. Neither cost is typically included in US cost-effectiveness analyses of vaccines in older populations [33], [34], nor in submissions to the Advisory Committee on Immunization Practices of the Centers for Disease Control and Prevention [35]. For these reasons, we did not include these costs in our base-case analysis.

However, because the target population in our study is likely to have relatively low labor force participation and high health care costs, the lives saved by the vaccination program may be relatively costly, in this sense, from a societal perspective. To assess the potential impact of including future costs for the additional survivors under the vaccination program, we first performed a sensitivity analysis that incorporated the age-specific annual per capita healthcare expenditure in the US estimated from a published study [36]. The year 2000 estimates for ages 65 and 85 were inflated to 2010 $US ($11,471 and $11,450, respectively) and the age-specific discounted yearly expenditures were applied to survivors over the remainder of their expected lifetime. Adding these future medical costs increases the ICERs from a societal perspective slightly to $340,000 at an incidence of 25/100,000 and to $21,000 at an incidence of 200/100,000. Considering total expenditures of $36,802 per year among US residents aged >65 estimated from the US Bureau of Labor Statistics [37], increases the ICERs to $349,000 and $30,000, respectively.

All estimates of costs and outcomes used in the model were derived and synthesized from a variety of data sources, and this process of interpretation and synthesis is subject to bias. We recognize that different assumptions may have yielded different results. We also note that model inputs were selected to reflect US epidemiology, and outcomes were calculated based on US costs life expectancy and age-specific utilities. Generalizing our results to other settings, in particular those with substantially lower health-care costs, should therefore be approached with caution.

Although no other published models have examined the cost-effectiveness of vaccinating the U.S. population aged ≥65 years, our findings are generally consistent with other studies that have examined the cost-effectiveness of pertussis vaccination in other age groups. For example Lee and colleagues evaluated both a one-time and decennial vaccination strategy in adults aged 20–64 years using a static Markov model and concluded that at disease incidences of greater than 120 cases per 100,000 populations, both strategies are cost-effective at a $50,000/QALY threshold [12].

A cost-effectiveness study of pertussis vaccination in adults aged ≥65 years conducted by the CDC reported a cost per QALY saved of $31,000 assuming an incidence rate of 104 cases/100,000 [11], while our study reports a cost/QALY of $63,000 per QALY gained at an incidence rate of 100 cases/100,000. Differences in base-case estimates, including vaccine efficacy, waning immunity, medical costs, and duration of illness, likely contributed to the difference in outcomes.

Because of the uncertainty and conflicting evidence about the true pertussis incidence, our conclusion is conditional upon the estimate of incidence. Using the best available efficacy and cost information, our static model analysis suggests that when pertussis incidence in adults ≥65 years is greater than 116 cases/100,000, replacing Td with *Tdap* yields a cost-effectiveness ratio less than $50,000/QALY gained from both the societal and payer perspectives. Estimates of pertussis incidence from other sources suggest that 116 cases/100,000 is a plausible estimate of pertussis incidence [11]. Vaccinating those aged 65 years yields a cost-effectiveness ratio similar to published estimates for adult vaccination [12], and the model yields relatively stable results under various scenario and sensitivity analyses.

Boostrix and Infanrix are trademarks of the GlaxoSmithKline group of companies. Adacel is a trademark of Sanofi-Pasteur.
